# Genetic insights into the dissolution of dioecy in diploid persimmon *Diospyros oleifera* Cheng

**DOI:** 10.1186/s12870-023-04610-3

**Published:** 2023-11-30

**Authors:** Peng Sun, Soichiro Nishiyama, Huawei Li, Yini Mai, Weijuan Han, Yujing Suo, Chengzhi Liang, Huilong Du, Songfeng Diao, Yiru Wang, Jiaying Yuan, Yue Zhang, Ryutaro Tao, Fangdong Li, Jianmin Fu

**Affiliations:** 1 State Key Laboratory of Tree Genetics and Breeding, Key Laboratory of Non-Timber Forest Germplasm Enhancement & Utilization of State Administration of Forestry and Grassland, Research Institute of Non-Timber Forestry, Chinese Academy of Forestry, Zhengzhou, 450003 China; 2 Graduate School of Agriculture, Kyoto University, Kitashirakawa Oiwakecho, Sakyo-Ku, Kyoto, 606-8502 Japan; 3 State Key Laboratory of Plant Genomics, Institute of Genetics and Developmental Biology, Chinese Academy of Sciences, Beijing, 100101 China

**Keywords:** Sexual diversity, Methylome, Transcriptome, Small RNAs, Genome-wide association study

## Abstract

**Background:**

Dioecy, a sexual system of single-sexual (gynoecious/androecious) individuals, is rare in flowering plants. This rarity may be a result of the frequent transition from dioecy into systems with co-sexual individuals.

**Results:**

In this study, co-sexual expression (monoecy and hermaphroditic development), previously thought to be polyploid-specific in *Diospyros* species, was identified in the diploid *D. oleifeara* historically. We characterized potential genetic mechanisms that underlie the dissolution of dioecy to monoecy and andro(gyno)monoecy, based on multiscale genome-wide investigations of 150 accessions of *Diospyros oleifera*. We found all co-sexual plants, including monoecious and andro(gyno)monoecious individuals, possessed the male determinant gene *OGI*, implying the presence of genetic factors controlling gynoecia development in genetically male *D. oleifera*. Importantly, discrepancies in the *OGI*/*MeGI* module were found in diploid monoecious *D. oleifera* compared with polyploid monoecious *D. kaki*, including no *Kali* insertion on the promoter of *OGI*, no different abundance of smRNAs targeting *MeGI* (a counterpart of *OGI*), and no different expression of *MeGI* between female and male floral buds. On the contrary, in both single- and co-sexual plants, female function was expressed in the presence of a genome-wide decrease in methylation levels, along with sexually distinct regulatory networks of smRNAs and their targets. Furthermore, a genome-wide association study (GWAS) identified a genomic region and a *DUF247* gene cluster strongly associated with the monoecious phenotype and several regions that may contribute to andromonoecy.

**Conclusions:**

Collectively, our findings demonstrate stable breakdown of the dioecious system in *D. oleifera*, presumably also a result of genomic features of the Y-linked region.

**Supplementary Information:**

The online version contains supplementary material available at 10.1186/s12870-023-04610-3.

## Background

Dioecy—male and female reproductive organs in separate individuals—is found in only approximately 6% of angiosperm species, but in diverse plant lineages [1–3]. This low rate of dioecy may indicate an evolutionary dead-end scenario [4]; however, it has been proposed that the dissolution of dioecy, which leads to re-evolution of the co-sexual systems, occurs during sexual-system evolution [5]. Multiple empirical studies have yielded results consistent with this hypothesis [6, 7].

**Fig. 1:**
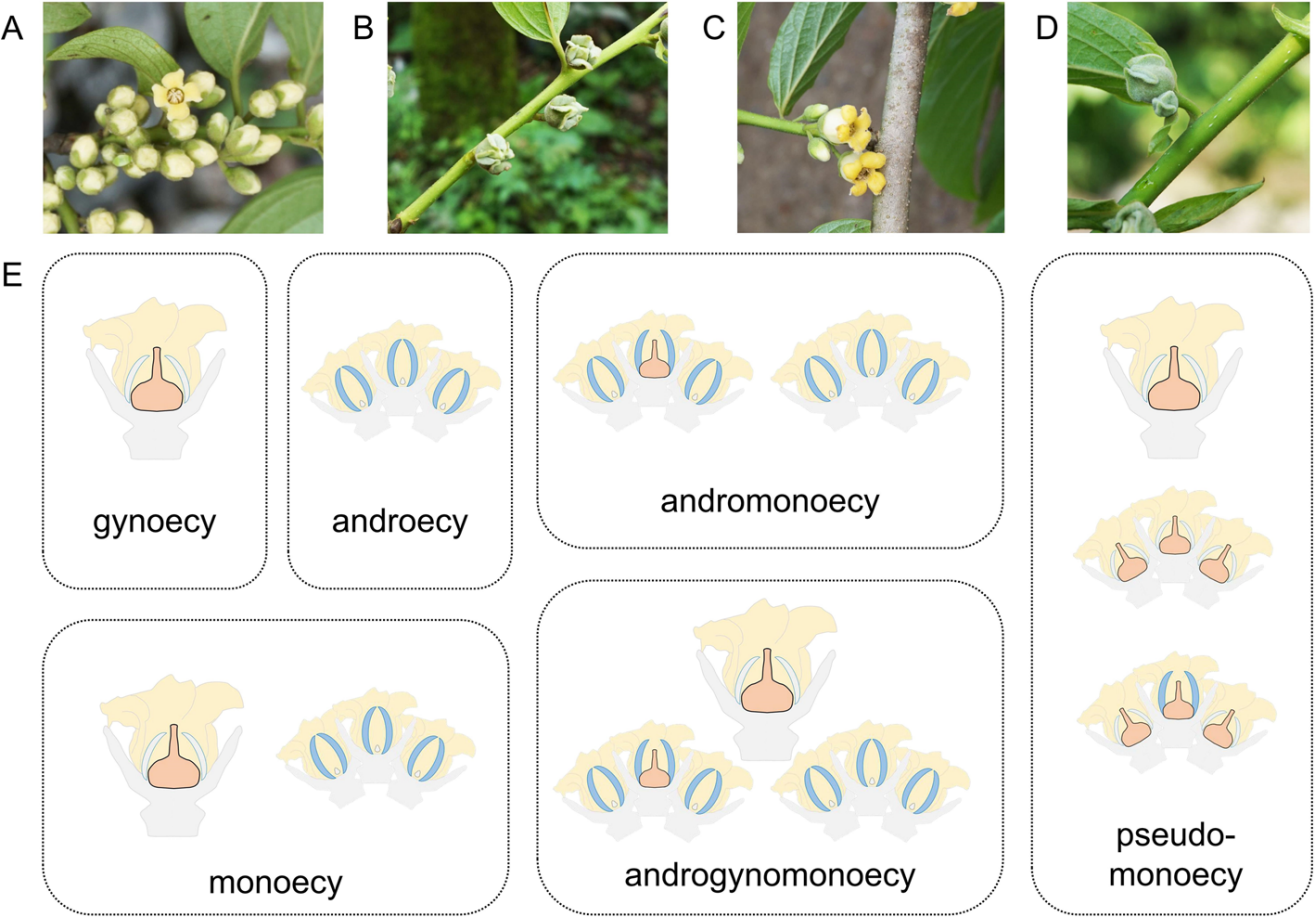
Diverse sex expressions in *D. oleifera*. **A** male floral buds, **B** female floral buds, **C** hermaphroditic floral buds, and **D** female flower cyme in pseudo-monoecy. **E** Schematic representation of individual sexual types. Details of the sexual types are shown in Figs. S1-3. Gynoecy and androecy represent female and male individuals in a dioecious population, respectively. Monoecy represents individuals bearing both female and male flowers. Andromonoecy represents individuals bearing both male and hermaphroditic flowers. Androgynomonoecy represents an individual bearing female, male and hermaphroditic flowers. Pseudo-monoecy represents individuals bearing two- or three-flower cymes with a female (or occasionally hermaphroditic) flower in the middle and one or two abnormal small female flowers at the sides

Sex expression in the genus *Diospyros* is diverse, and several key genetic components controlling sexual expression have been identified [8]. Diploid *D. lotus* is dioecious, exhibiting separate male and female plants; polyploid *D. kaki* encompasses gynoecious (female flower only), androecious (male flower only), monoecious (male and female flowers), androgynomonoecious (male, female, and hermaphroditic flowers), and andromonoecious (male and hermaphroditic flowers) individuals [9–12]. Similar sexual diversity was observed in diploid *D. oleifera* (Fig. 1; Figs. S1-3). In *Diospyros*, male flowers are typically observed in the form of a three-flower cyme, but female flowers are always solitary (Fig. 1). Dioecy is considered an ancestral form in *Diospyros*; therefore, it is reasonable to assume that the polygamous system in *D. kaki* and *D. oleifera* evolved from the dioecious system [13]. In diploid dioecious *D. lotus*, the small RNA (smRNA)-coding gene *OGI* determines the formation of male trees via repression of the feminising gene *MeGI* [8]. In contrast, an additional layer of regulation in the form of DNA methylation of the *MeGI* promoter may contribute to monoecy in hexaploid *D. kaki* [10].

Although several important molecular mechanisms underlying the sexuality determination of *D.* spp. have been identified, the fundamental molecular mechanisms that underlie the sexual diversity of some *D.* spp. (*e*.*g*., *D. kaki* and *D. oleifera*) are unclear [14]. In this study, we used a genetic approach to characterise a *D. oleifera* population. The diploidy of *D. oleifera* with all types of sex expressions was confirmed (Method S1; Text S1; Fig. S4). The genome of diploid *D. oleifera* is simpler than the genome of cultivated polyploid *D. kaki*, and a new *D. oleifera* genome was assembled at the chromosome scale (Text S2; Figs. S5-S17; Tables S1-S12) [15]. The development of *D. oleifera* floral buds is synchronous to the development of *D. kaki*. Therefore, the analysis of *D. oleifera* could be a novel addition for deeper understanding of sex diversity in *Diospyros*.

In mid-April 2019 and 2021, we surveyed the sex phenotype of *D. oleifera* obtained from a natural population in Guilin City, Guangxi Zhuang Autonomous Region, China (25''04′46.08–25''56′14.00 N; 110''17′57.65–111''03′23.98 E) (Fig. S18). Two hundred eight plants with various types of sex expressions were sampled in total (Table 1; Table S13). The genetic components of the *OGI*/*MeGI* system were examined in the *D. oleifera* population. Subsequently, comparative-methylome and transcriptome analyses were performed to identify the molecular mechanism responsible for sexual diversity. Moreover, a genome-wide association study (GWAS) was conducted to determine candidate genomic regions and genes that contribute to the sexual diversity.

**Table 1:** Sex expressions in a *D. oleifera* natural population

Individual sexual types	Number	DNA sampled	*OGI* genotype		Male expression
			*OGI*^+^	*OGI*^−^	
Gynoecy	116	58	4	54	-
Pseudo-monoecy	2	2	0	2	-
Androecy	45	45	45	0	+
Monoecy	28	28	27	1	+
Androgynomonoecy	10	10	10	0	+
Andromonoecy	7	7	7	0	+
Total	208	150	93	57	

## Results

### Characterisation of the male-specific region (the MSR) in D. *oleifera*

We analysed the presence of the MSR [16] and the *OGI* gene in 150 *D. oleifera* individuals (Table 1; Table S13). We found that 54 of 58 gynoecious individuals did not contain the MSR or *OGI* (*OGI*-negative, *OGI*^−^). In contrast, all 45 androecious individuals contained the MSR and OGI (*OGI*-positive, *OGI*^+^). All co-sexual plants (monoecious, androgynomonoecious, and andromonoecious individuals), except for one monoecious individual, contained the MSR and were *OGI*^+^; these findings strongly suggested that *OGI* is required for male tissue production in general sex determination of *D. oleifera*, as in monoecious *D. kaki* [17]. Among the co-sexual plants, the ability to produce male flowers was variable (Table S13), and thus the four exceptional gynoecious plants possessing *OGI* were considered female-biased monoecious plants. We did not include plants with inconsistent phenotypes (four gynoecious with *OGI*) in subsequent analyses.

Several individuals showed distinct flower sexes and morphologies, which comprised pseudo-monoecy. The pseudo-monoecious trees, lacking the MSR and *OGI*, form two- or three-flower cymes with a female (or occasionally hermaphroditic) flower in the middle and one or two abnormal small female flowers at the sides (Fig. 1D; Fig. S3).

**Fig. 2:**
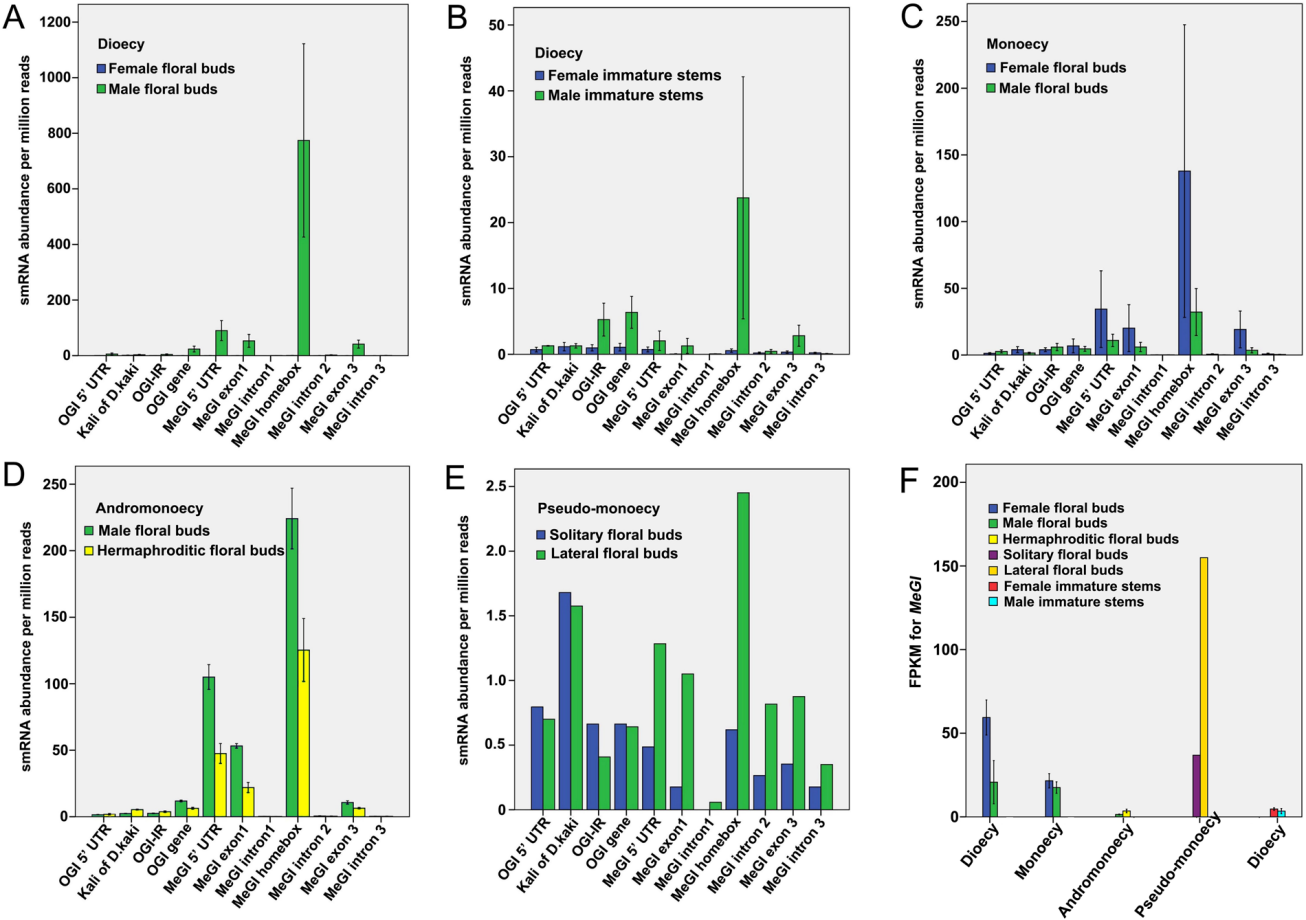
*MeGI* expression and smRNA accumulation in *D. oleifera*. **A**-**E** smRNA accumulation on *OGI*/*MeGI* genomic sequences in (**A**) female and male floral buds in dioecy; **B** stems of female and male shoots in dioecy; **C** female and male floral buds in monoecy; **D** male and hermaphroditic floral buds in andromonoecy; and **E** solitary floral buds and lateral floral buds of flower cymes in pseudo-monoecy. **F** Fragments per kilobase of transcript per million mapped reads (FPKM) values of *MeGI* in floral buds and stems. Data are means ± standard errors (three biological replicates) except for floral buds in pseudo-monoecious trees, for which no biological replicates were available

In *D. kaki*, the *OGI* mRNA expression levels in both female and male floral buds of *D. kaki* were very low during development, which was attributed to the presence of *Kali* (a short interspersed nuclear element [SINE]-like insertion) on the *OGI* promoter [10]. However, in this study, none of the *OGI*^+^ *D. oleifera* individuals harboured *Kali* on the promoter of *OGI* (Method S5; Fig. S19). *OGI* is a pseudo-gene, which encodes small RNAs (smRNAs) [8]. Thus, the accumulation patterns of smRNAs on *OGI* gene were used to represent the *OGI* expression levels here. The abundance of smRNAs on *OGI* gene was significantly higher in male floral buds obtained from all the androecious, monoecious and andromonoecious *D. oleifera* trees (*OGI*^+^ individuals) than that in the female floral buds obtained from gynoecious trees (*OGI*^−^ individuals) (Fig. 2A, C and D), which suggest that *OGI* is not seriously suppressed in *D. oleifera*. Therefore, previous molecular genetic knowledges for monoecious production in *D. kaki* might be not directly applicable to *D. oleifera*.

### Different profiles of smMeGI abundance and MeGI transcripts between single-sexual and co-sexual D. *oleifera*

We investigated the accumulation patterns of smRNAs on *OGI* and *MeGI* regions (*smMeGI*) in *D. oleifera* (Fig. S19A); such patterns coincide with flower sex in *D. lotus* and *D. kaki* [8, 10]. In the single-sexual (gynoecious and androecious plants) *D. oleifera*, greater accumulation of *smMeGI* was detected in floral buds from androecious plants (androecious male; A_M) than in floral buds from gynoecious plants (gynoecious female; G_F) (Fig. 2A), similar to findings in *D. lotus* and *D. kaki* [10]. A similar pattern was observed in the immature stem tissues of flowering shoots (Fig. 2B). *smMeGI* degrade *MeGI* transcripts, thus as an expected consequence of the level of *smMeGI*, the level of *MeGI* transcripts was significantly higher in G_F than in A_M in mid-April (Fig. 2F), which was in accordance with the patterns in *D. lotus* and *D. kaki* [8, 10].

In contrast, the *smMeGI* patterns in the co-sexual types were distinct from the findings in previous reports. In the monoecious *D. oleifera*, where individuals exhibited separate male and female flowers, *smMeGI* accumulation was not significantly different between the female (monoecious female; M_F) and male floral buds (monoecious male; M_M) (Fig. 2C). Similarly, the level of *MeGI* expression was not significantly different between M_F and M_M; it was comparable with the level in male flower buds of androecious plants (A_M) (Fig. 2F).

In the hermaphroditic flowers, we observed an intermediate level of *smMeGI*. Among andromonoecious plants, the level of *smMeGI* accumulation was significantly lower in hermaphroditic floral buds (andromonoecious hermaphroditic; AM_H) than in male floral buds (andromonoecious male; AM_M) (Fig. 2D). In contrast to female flowers (G_F), where *smMeGI* was nearly absent, a substantial amount of *smMeGI* accumulation was detected in hermaphroditic flowers (AM_H) (Fig. 2A and D). The levels of *MeGI* expression were not significantly different between AM_M and AM_H (Fig. 2F).

In the *OGI*^―^ pseudo-monoecious plants, low *smMeGI* accumulation was observed in both solitary female floral buds (pseudo-monoecious solitary female; PM_SF) and lateral floral buds of the three flower cymes (pseudo-monoecious lateral female; PM_LF) (Fig. 2E). *MeGI* expression was higher in PM_SF and PM_LF than in male floral buds from androecious, monoecious, and andromonoecious plants, consistent with the level of gynoecia development (Fig. 2F).

### DNA methylation in D. *oleifera* floral buds and stems

In monoecious *D. kaki*, a lower methylation level of the *MeGI* promoter is associated with female flower formation in genetically male plants [10]. We evaluated whether this mechanism is active in the co-sexual *D. oleifera*, where sex expression does not match with the component of *OGI*/*MeGI* system. The DNA methylation levels of floral buds and stems were analysed by whole-genome bisulphite sequencing (Text S3; Tables S14-S19; Figs. S20-S21).

**Fig. 3:**
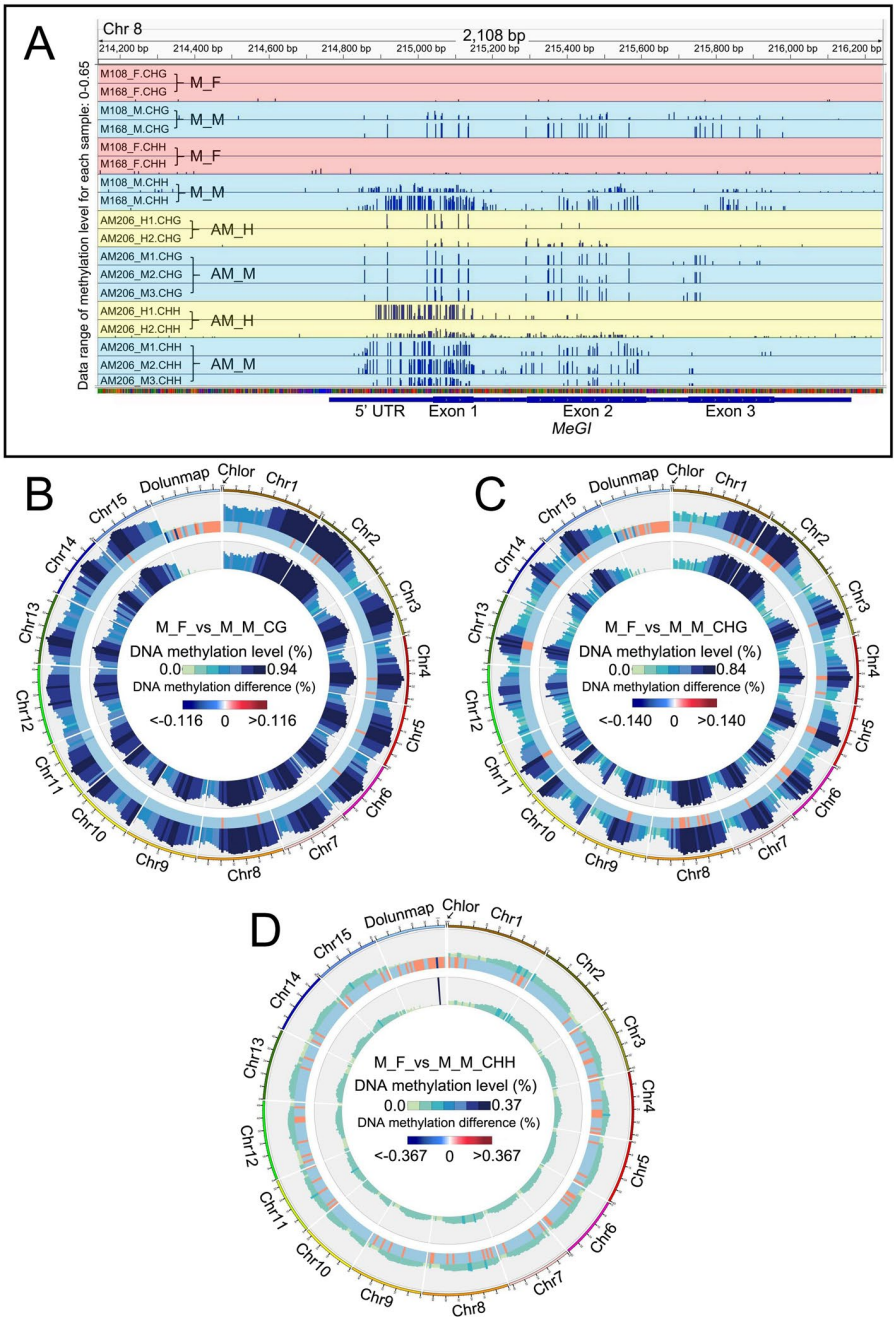
DNA methylation in flower buds from co-sexual *D. oleifera*. **A** Methylation levels of the *MeGI* genomic region in floral buds from monoecious and andromonoecious *D. oleifera*. **B**-**D** Whole-genome comparison of methylation levels in the **B** CG, **C** CHG, and **D** CHH subcontexts between M_F and M_M. Tracks from outside to inside: methylation level of M_F; different methylation levels between M_F and M_M, where red and blue represent higher and lower methylation levels in M_F than in M_M, respectively; methylation level of M_M. Dolunmap and Chlor in (**A**), (**B**) and (**C**) represent the male-unmapped sequences and chloroplast genome, respectively

Unexpectedly, the DNA methylation patterns of the *MeGI* region in diverse *D. oleifera* matched the DNA methylation patterns of *D. kaki* and *D. lotus*, even in the co-sexual types. The methylation levels of the 5’-untranslated region and exon of *MeGI* were significantly lower in female floral buds than in male floral buds obtained from monoecious plants (Fig. 3A). This pattern was also observed in andromonoecious and single-sexual (gynoecious and androecious individuals) plants, which showed lower methylation levels in female tissues (AM_H, G_F, and G_S) than in male tissues (AM_M, A_M, and A_S) (Fig. 3A; Figs. S22 and S23).

We next speculated that the discrepancy between the *smMeGI* level and DNA methylation in the co-sexual plants is related to changes in the global methylation pattern. Notably, the DNA methylation levels of floral buds in the CG, CHG, and CHH subcontexts were lower in female tissues (M_F) than in male tissues (M_M) in almost all genomic regions (Fig. 3B, C, and D). The DNA methylation levels in the CG and CHG subcontexts in every part of gene body, as well as the surrounding regions, were generally lower in M_F than in M_M (Fig. S24A and B). Most of the differentially methylated regions were distant from genes, although there were numerous differentially methylated regions in the promoter, exon, and intronic regions (Fig. S24C, D, and E). A similar pattern was observed in the single-sexual plants; the genome-wide DNA methylation levels in the CG, CHG, and CHH subcontexts were generally lower in females (G_F) than in males (A_M) (Fig. S25). The same trend was observed in stems of flowering shoots (Fig. S26). This finding was also observed for hermaphrodite flower formation. In andromonoecious plants, the DNA methylation levels of floral buds in all three subcontexts were lower in hermaphrodites (AM_H) than in males (AM_M) (Fig. S27). Therefore, female tissues showed lower global DNA methylation levels than male tissues in all sexual types, implying that a genome-wide decrease in DNA methylation promotes the development of gynoecia in both co- and single-sexual systems.

As a potential regulator of DNA methylation, we investigated the expression levels of DNA methyltransferase/demethylase genes by RNA-Seq. A demethylase gene (evm.model.Chr7.1501), homologous to REPRESSOR OF SILENCING1 (*ROS1*) [18], was downregulated in AM_M compared with AM_H (Table S20), possibly explaining the dynamic modulation of genome-wide methylation levels in andromonoecious *D. oleifera*.

### Overlap of mRNA-miRNA functional modules between co- and single-sexual systems

To characterise the functional overlap of gynoecia/androecia development in different sexual expression systems, transcriptome analyses were conducted using 26 samples of floral buds and stems of flowering shoots (Text S4; Figs. S28-S37; Tables S21-S26). We found common sexually distinct regulatory networks of microRNAs (miRNAs) and their targets, as well as common functional enrichments for gynoecia development in single- and co-sexual systems.

**Fig. 4:**
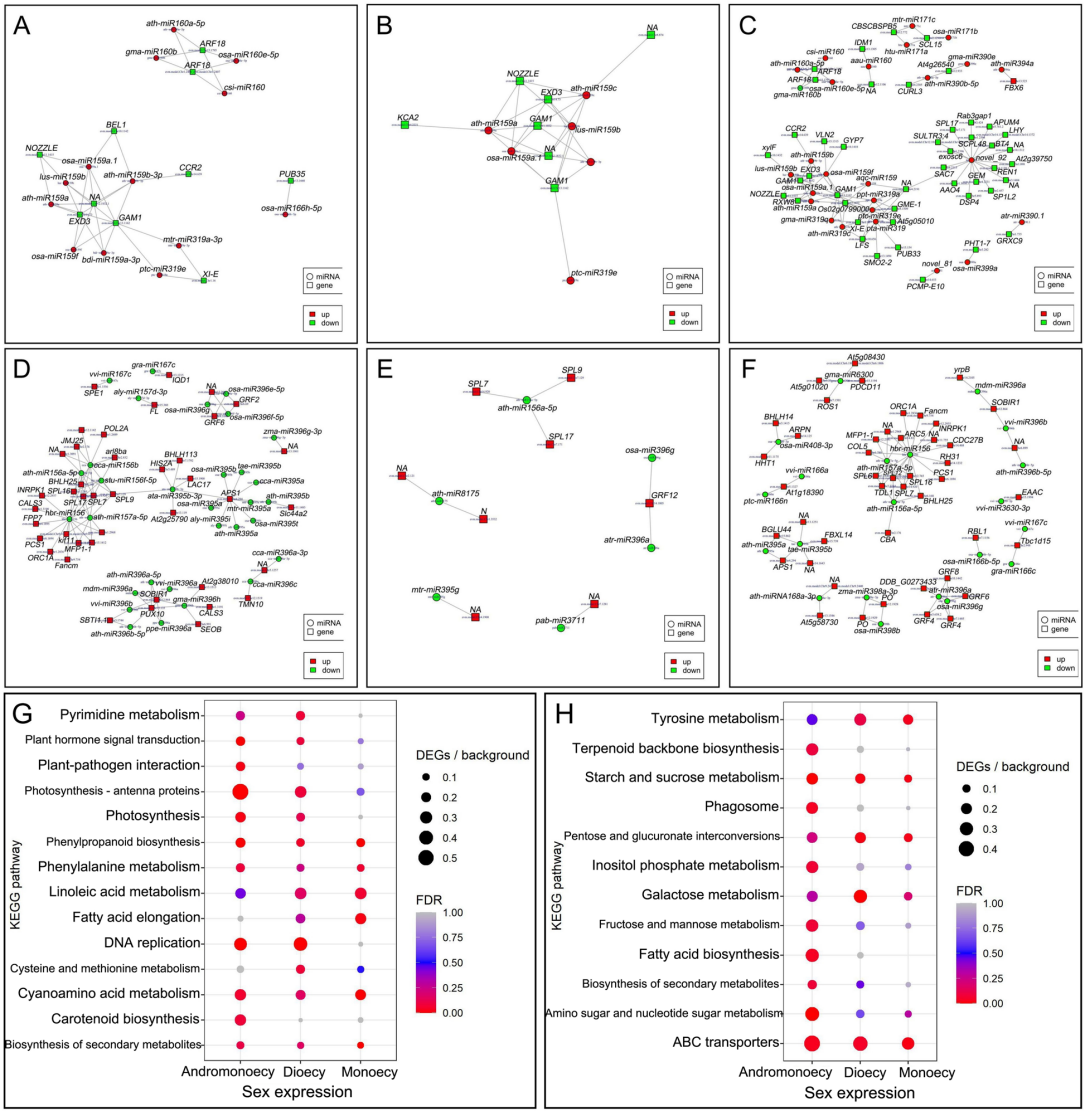
Networks constructed based on differentially expressed miRNAs and their mRNA targets, as well as KEGG pathway enrichments of differentially expressed genes. miRNAs with higher expression and their downregulated targets (**A**) in G_F compared with A_M, (**B**) in M_F compared with M_M, (**C**) in AM_H compared with AM_M, (**D**) in A_M compared with G_F, (**E**) in M_M compared with M_F, and (**F**) in AM_M compared with AM_H. KEGG pathway enrichments [19–21] of (**G**) up- and (**H**) downregulated genes in female (or hermaphroditic) floral buds compared with male floral buds

Based on the interaction network analysis and functional annotation, highly expressed miRNAs in female (or hermaphroditic) and their mRNA targets downregulated in female (or hermaphroditic) floral buds, and vice versa (*i.e.,* low-level miRNA and high-level mRNA targets in females), were identified in single- and co-sexual systems (Fig. 4; Tables S25 and S26). At least two of the three male-active networks (low-level miRNA and high-level mRNA targets in male tissues) (Fig. 4A, B, and C) included the exonuclease mut-7 homolog, *NOZZLE*, *GAMYB* (*GAM1*), auxin response factor 18 family members, cinnamoyl-CoA reductase 2, myosin-11, and evm.model.Chr12.1832.1. Specifically, *NOZZLE* and *GAM1* were detected in all three networks. In contrast, at least two of the three female-active networks (low-level miRNA and high-level mRNA targets in female tissues) (Fig. 4D, E, and F) included squamosa promoter-binding-like protein gene* 7* (*SPL7*), *SPL9*, *SPL16*, *SPL17*, lysine-specific demethylase (*JMJ25*), *BHLH25*, *PCS1*, MAR-binding filament-like protein 1–1 (*MFP1-1*), origin of replication complex subunit 1A (*ORCS-1A*), Fanconi anaemia group M protein homolog, and cucumisin, growth-regulating factor 6 (*GRF6*), *ORCS-1A*, ATP sulphurylase 1-chloroplastic, and *SOBIR1*.

Comparisons of female (or hermaphroditic) and male tissues revealed that numerous Kyoto Encyclopedia of Genes and Genomes (KEGG) pathways were commonly enriched in both single- and co-sexual systems, including the female-active pathways phenylpropanoid biosynthesis, biosynthesis of secondary metabolites, and plant hormone signal transduction, etc.(Fig. 4G), as well as the male-active pathways starch and sucrose metabolism and galactose metabolism, etc.(Fig. 4H) [19–21]. Therefore, it was suggested that single- and co-sexual systems use the same basic functional modules for flower sex determination.

### Core gene networks correlated with sex differentiation in female and male floral buds

To evaluate the regulatory paths of sex differentiation in female and male floral buds in co- and single-sexual systems, coexpression patterns were visualised by weighted correlation network analysis using all female and male floral buds that had been subjected to RNA-Seq analysis (Table S27). A scale-free topology model fit soft threshold of 7 (Fig. S38A and B) was applied and the coexpression pattern was clustered into 21 modules (Fig. S38C and D).

The midnight-blue and yellow modules showed the strongest positive correlations with male expression, and genes in these modules may promote the development of male floral buds (Fig. S38E). The midnight-blue module contained 156 genes, which were significantly (corrected *P* < 0.05) enriched in five GO terms: regulation of transcription DNA-templated, regulation of primary metabolic process, regulation of gene expression, regulation of cellular macromolecule biosynthetic process, and copper ion binding (Fig. S39A). The yellow module contained 378 genes, which were significantly enriched in the organic substance transport GO term (Fig. S39B).

The pink and green modules showed the strongest positive correlations with female expression, and genes in these modules may promote the development of female floral buds. The pink module contained 285 genes, which were significantly enriched in five GO terms: UDP-glycosyltransferase activity, transferase activity, transferring glycosyl groups, single-organism process, single-organism metabolic process, and oxidoreductase activity (Fig. S39C). Genes in the pink module were also significantly enriched in two KEGG pathways: phenylpropanoid biosynthesis and linoleic acid metabolism (Fig. S39D).

**Fig. 5:**
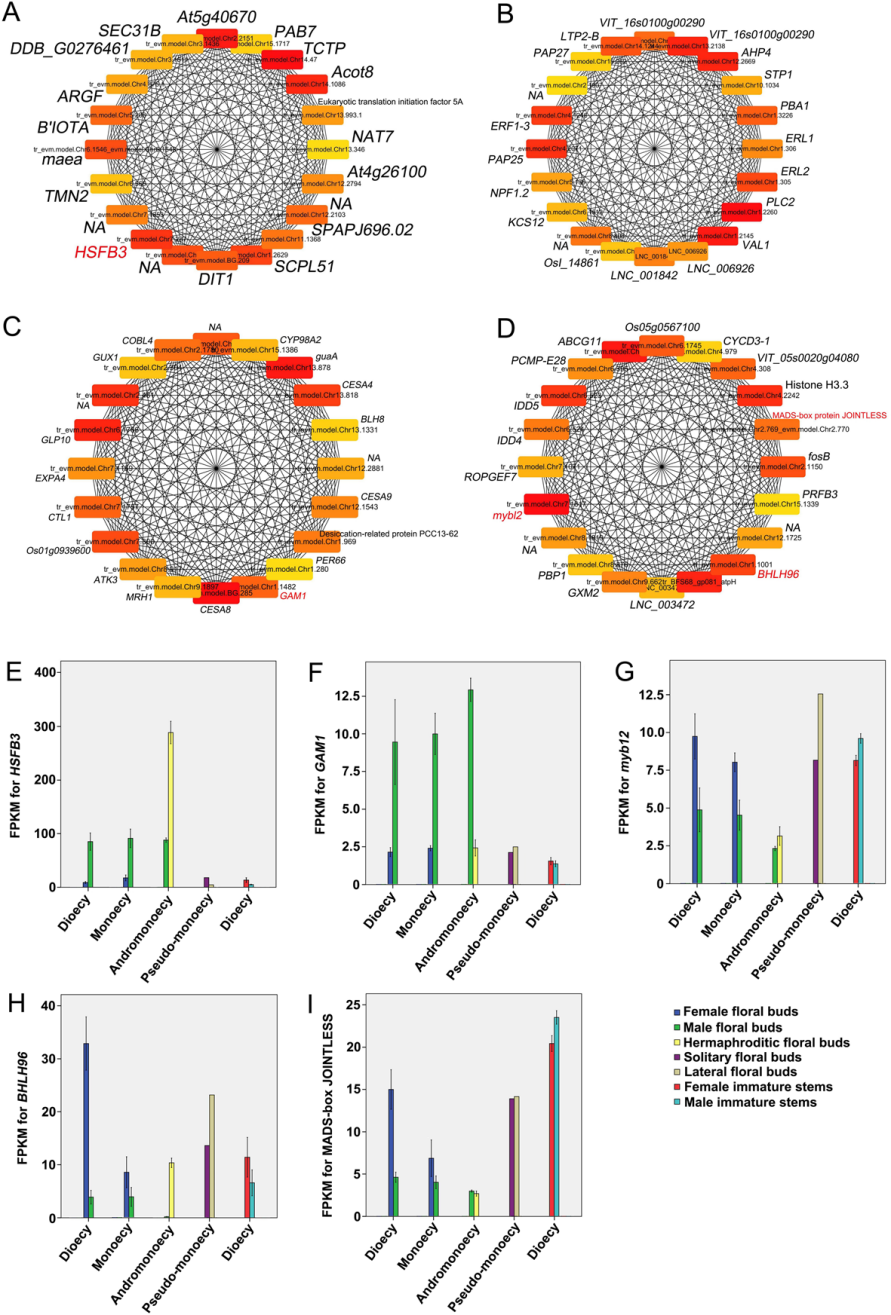
Networks constructed with genes harbored top 20 connections in the (**A**) midnight-blue, (**B**) yellow, (**C**) pink, and (**D**) green modules, respectively. FPKM values of (**E**) *HSFB3,* (**F**) *GMA1,* (**G**) *myb12,* (**H**) *BHLH96* and (**I**) MADS-box JOINTLESS in floral buds and stems. Gene names in red in (**A**), (**C**) and (**D**) represent TFs. The rectangle color from dark red to light yellow in (**A**-**D**) represents a decreasing trend of connectivity. Data are means ± standard errors (three biological replicates) except for floral buds in pseudo-monoecious trees, for which no biological replicates were available in (**E**-**I**)

Genes with top 20 connections were identified in the midnight-blue, yellow, pink, and green modules, and used to construct networks, respectively (Fig. 5). Transcription factors (TF) in the networks were highlighted. Heat stress TF B-3 (*HSFB3*) in the midnight-blue module and B3 domain-containing transcription repressor (*VAL1*) in the yellow module were shown to be potential key genes for male development (Fig. 5A and B). *HSFB3* showed sharply higher expression levels in male floral buds than in female in both dioecious and monoecious plants (Fig. 5E), supporting a potential male promoting effect. Although *GAM1* was identified in the pink module which was positively correlated with female development, the expression levels of *GAM1* were slightly higher in male floral buds than that in female (Fig. 5F), suggesting this gene may promote male development. This is in accordance with the results shown in the mRNA-miRNA functional modules mentioned above. MADS-box protein JOINTLESS (*J*), Myb-related protein B (*myb12*) and bHLH96 (*BHLH96*) in the green module were shown to be potential key genes for female development (Fig. 5 C and D), which was further demonstrated by the results that all these genes were higher expressed in the female floral buds than in male in both dioecious and monoecious plants (Fig. 5G, H and I).

### Population structure in D. *oleifera* according to sexual expression

**Fig. 6:**
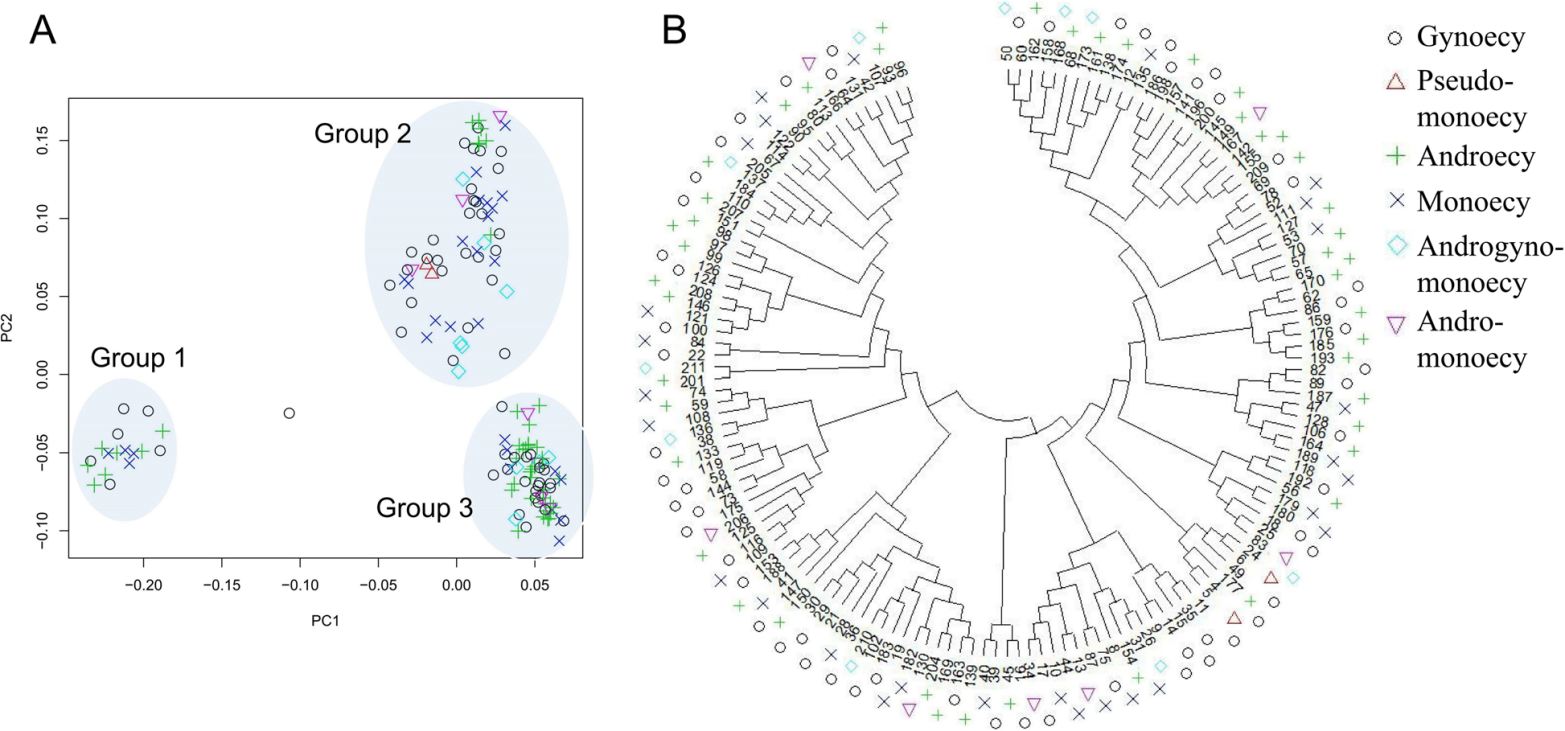
Phylogeny of the 150 *D. oleifera* plants. **A** Scatter clustering diagram based on the first two principal components after PCA of whole-genome sequence data. PC1 and PC2 explained 7.42% and 5.59% of the total variance, respectively. **B** Maximum-likelihood phylogenetic tree of the 150 *D. oleifera* plants using MEGA-X labelled in order of sampling time. Therefore, similar numbers indicate relatively close distributions

We performed whole-genome sequencing of 150 *D. oleifera* individuals with a mean depth of 20 × . After filtering, 3,545,359 single nucleotide polymorphisms (SNPs) and 318,863 indels were obtained for further analysis. The mean PI_HAT value was 0.056, indicating a low level of familial relationship in the population. PCA showed that the sampled individuals could be divided into three clusters (Fig. 6A). Single-sexual plants and monoecious plants were found in all three clusters, whereas andromonoecious and androgynomonoecious trees were found only in groups 2 and 3. Pseudo-monoecious plants were found only in group 2, and they are distributed in close proximity. The results imply that the monoecious genetic factor prevails in *D. oleifera*, whereas andromonoecious, androgynomonoecious, and pseudo-monoecious individuals may be rare and develop only under certain conditions. The maximum-likelihood phylogenetic tree supported this notion; individuals obtained in close areas tended to have a close genetic relationship (Fig. 6B). The decay of linkage disequilibrium (LD) with physical distance between SNPs occurred at < 200 bp (r^2^ = 0.2) (Fig. S40A).

### GWAS of the co-sexual phenotypes

To identify the genomic factors that confer the monoecious phenotype, we performed GWAS for the proportion of female shoots in genetically male plants. Ninety individuals with male flower production were selected; and 3,502,197 SNPs and 311,512 indels were retained for further analysis after LD pruning (Table S28).

**Fig. 7:**
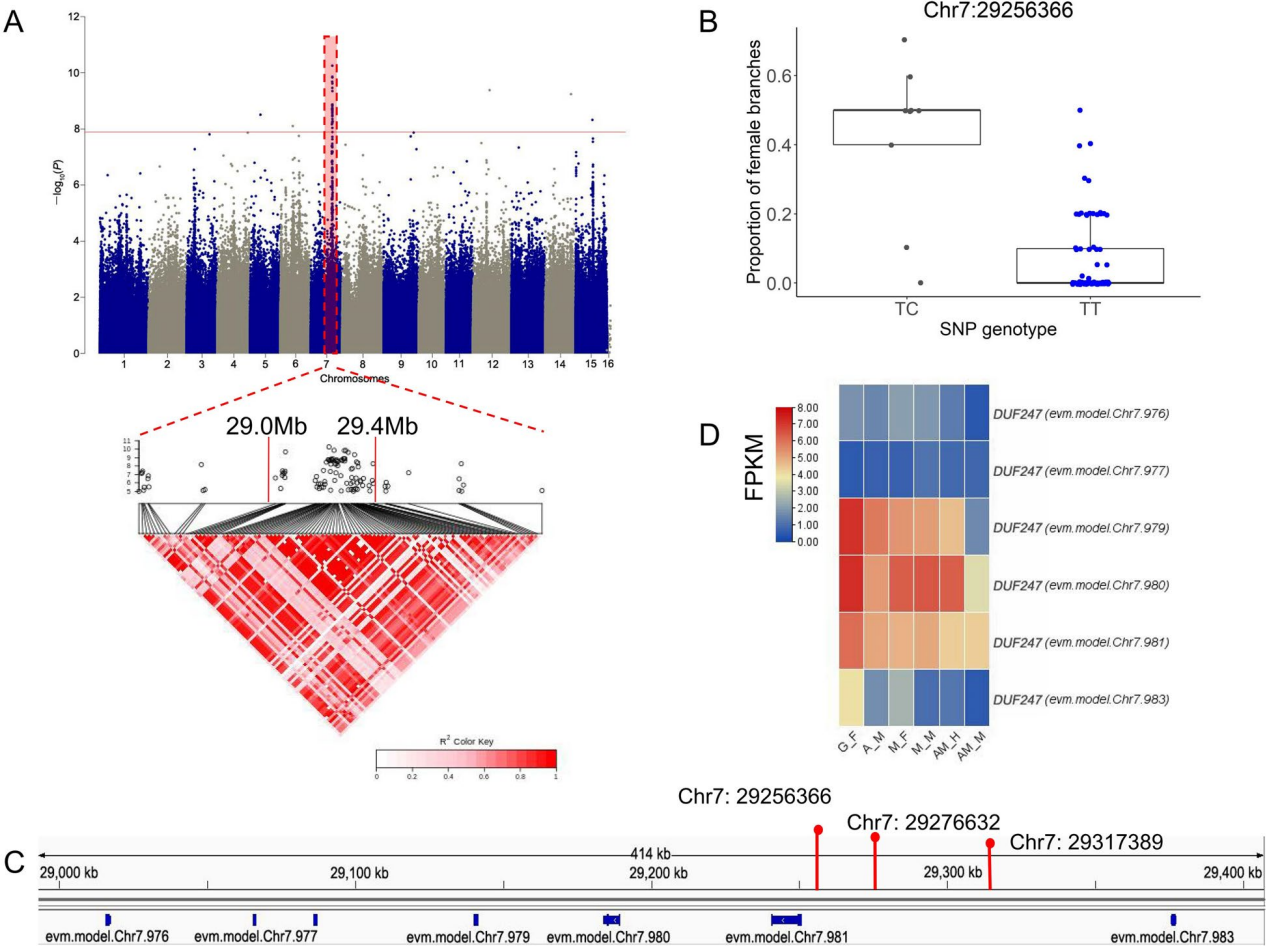
GWAS for the monoecious phenotype in *D. oleifera*. **A** Manhattan plot for the proportion of female shoots in 90 plants with male flower production (top), along with a local Manhattan plot and LD heatmap (bottom) of the associated region on chromosome 7. **B** Proportion of female shoots based on genotype at the most significant locus, Chr7: 29256366. **C** Schematic representation of gene position in the 29.0–29.4 Mb region of Chr7. **D** Expression pattern of genes in the 29.0–29.4 Mb region of Chr7 in female (or hermaphroditic) and male floral buds in single- and co-sexual systems. Red line in (**A**) represents the Bonferroni-corrected *P*-value of 0.05, as shown in Figs. 7A and 8A. Chromosome 16 in (**A**) represents the male-unmapped sequences, which is consistent in the following figures

GWAS for the proportion of female shoots (Table S13) detected a significant peak on chromosome 7 (Fig. 7A and S40B). Individuals with a heterozygous genotype at the locus with the strongest association signal showed greater proportions of female shoots (Fig. 7B; Table S29). A haplotype block spanning 29.0–29.4 Mb on chromosome 7 was strongly associated with phenotype (Fig. 7A). Seven genes of the *DUF247* family were identified in this block (Fig. 7C). Most were upregulated in female tissue compared with male tissue; one of these genes, evm.model.Chr7.983, was significantly upregulated in female floral buds compared with male floral buds in both monoecious and single-sexual plants (Fig. 7D; Table S30). Most of the variants with the highest peak association were distributed upstream of evm.model.Chr7.983 (Fig. 7C), which may contribute to the differential expression of this gene. miRNA pab-miR3711, located within the block spanning 29.0–29.4 Mb, was downregulated in female floral buds compared with male tissues in monoecious plants (Table S31), possibly in relation to monoecious phenotype development.

**Fig. 8:**
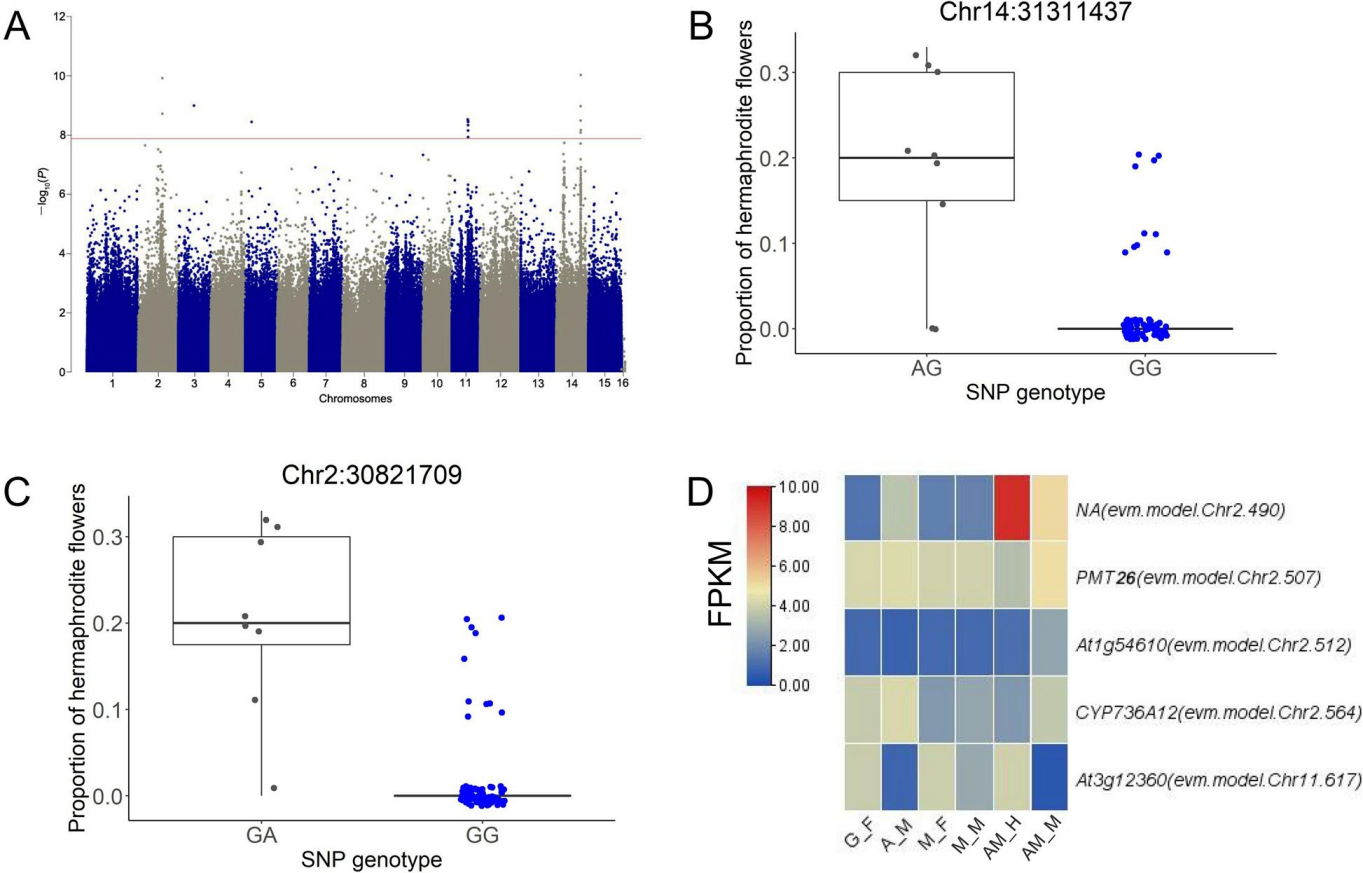
GWAS for hermaphroditic flower development in *D. oleifera*. **A** Manhattan plot for the proportion of hermaphroditic shoots among 90 samples with male flower production. **B** Proportion of hermaphroditic shoots based on genotype at the most significant locus, Chr14: 31311437, and **C** the second most significant locus, Chr2: 30821709. **D** Genes with significantly different expression in AM_M compared with AM_H in the association regions

We also recorded the proportion of hermaphroditic floral branches in the 90 plants. GWAS analysis for the proportion of hermaphrodite shoots revealed strong signals on chromosomes 2, 11, and 14 (Fig. 8A). According to the LD analysis, five, two, and three LD blocks with strong associations were detected on chromosomes 2, 11, and 14, respectively (Fig. S41). Individuals with heterozygous genotypes at the loci with the strongest association signals showed greater proportions of hermaphroditic shoots for all detected blocks (Fig. 8B and C; Table S32). In total, 65 genes were identified on the blocks, among which 3 and 2 genes were up- and downregulated in male floral buds compared with hermaphroditic floral buds in andromonoecious plants, respectively (Fig. 8D; Table S33). Additionally, genes encoding 31 lncRNAs (Table S34), and 9 miRNAs (Table S35) were identified in these regions. Further analysis of these sequences may identify genetic events linked to hermaphroditic flower development in genetically male *D.* spp.

### Absence of the YY genotype in the D. *oleifera* population

**Fig. 9:**
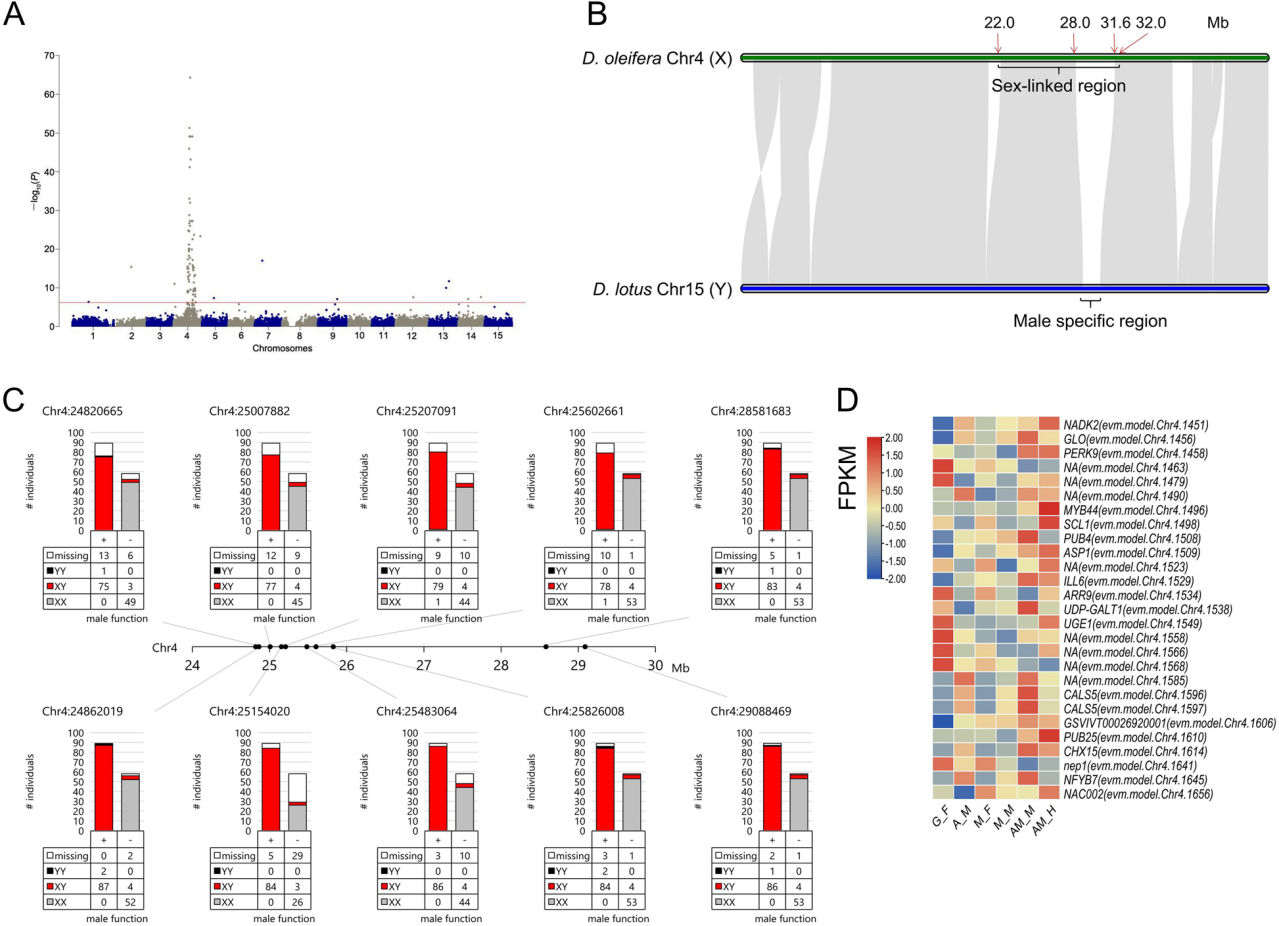
Genetic characterisation of the sex-linked region in the *D. oleifera* population. **A** GWAS for male expression. **B** Alignment of Chr4 of *D. oleifera* and Chr15 of *D. lotus*. **C** Genotype fractions of the SNPs with strong association signals in the sex-linked region. **D** Differentially expressed genes in the sex-linked region

The emergence of co-sexual phenotype enables crossing among genetically male plants. A model-based analysis showed that the stability of dissolution of dioecy depends on the viability of the YY genotype [22], which is reduced by loss of function of Y-linked genes. Therefore, we characterised the sex-linked region. GWAS for the male expression (or *OGI*^+^) (Table S13) of 150 plants identified a sex-linked region at 22.0–32.0 Mb on chromosome 4 (Figs. 9A and S42-43; Table S36). This region corresponds to the male-specific region in *D. lotus* [16] (Fig. 9B). The genotype of the SNPs with the strongest association signals in this region showed that most of the 54 gynoecious individuals (*OGI*^−^) were homozygous (XX type), whereas most of the 89 male-functional individuals were heterozygous (XY type) (Fig. 9C; Table S37). Genotyping analysis indicated that the YY genotype was not supported by > 2 successive variant loci (Table S41). The occasional YY genotype may be attributed to recombination or genotyping error associated with the highly repetitive nature of this region. Therefore, the YY genotype is either present at a negligible level or absent from the population.

We detected a potential X-specific region and putative sex-related genes in this region, in addition to the male determinant *OGI* (Text S5; Table S38). Twenty-seven genes were differentially expressed between female (or hermaphroditic) and male floral buds in single- or co-sexual systems, or both (Fig. 9D). Specifically, two genes, *GLO* and *ARR9*, which have masculinising functions in *Antirrhinum majus* [23] and feminising functions in the genus *Populus* [24], respectively, were differentially expressed in this region (Fig. 9D).

## Discussion

### A sex-linked region contributing to sex dimorphism and diversity

All *D. oleifera* trees with male function, but exceptional one monoecious individual, had the MSR and *OGI* (Table 1), and they showed many heterozygous genotypes in the sex-linked region on chromosome 4 (Fig. 8), consistent with the male heterogametic (XY) system. *smMeGI* and *MeGI* transcript analyses implied that the *OGI*/*MeGI* system [8] functions in the sex determination of single-sexual *D. oleifera* plants (Fig. 2).

In the *D. oleifera* population, two types of co-sexual systems were identified: monoecy and hermaphroditic flower formation. The genotyping results (Table 1) indicate that all individuals can be divided into two groups: a genetically female (XX) group that includes gynoecious and pseudo-monoecious plants, and a genetically male (XY) group that includes androecious, monoecious, androgynomonoecious, and andromonoecious plants. Thus, the co-sexual types (monoecious, androgynomonoecious, and andromonoecious) may represent results of the breakdown of dioecy, where androecious trees acquire gynoecia development functions as demonstrated empirically in various species, including grape and papaya [5, 22, 25–27].

The lack of YY individuals in this study (Fig. 8) was surprising to us because it was inconsistent with the potential for crossing among genetically male individuals, considering that flowering times of male and female flowers in *D. oleifera* (and *D. kaki*) usually fully overlapped. This finding may be attributed to the low viability of the YY genotype (*i.e.,* genetic degeneration). Genetic modelling has shown that under such conditions, stable coexistence of single- and co-sexual plants in a population (subdioecy) can be achieved, because it is likely that Y chromosome has not yet evolved to fully suppress female functions [22]. In the present study, genomic and transcriptional analyses identified putative Y-/X-specific regions (Fig. 8C), as well as several genes in the sex-linked region that are differentially expressed between female and male tissues and may function in sexual expression, such as *GLO* and *ARR9* (Text S5); these findings imply functional divergence of the X and Y chromosomes. *GLO* is reportedly essential for stamen development in *Antirrhinum majus* [23], whereas *ARR* proteins function in gynoecia development and are regarded as master regulators of sex expression in the genus *Populus* [24]. The sex-linked region and the essential genes within that region presumably cooperate with the *OGI*/*MeGI* system to regulate the sex differentiation and diversity of *D*. spp.

### Feminising scenario that contribute to the dissolution of dioecy in co-sexual D. *oleifera*

We identified three potential feminising mechanisms in both single- and co-sexual systems: increased expression of the feminising gene *MeGI* and decreased abundance of *smMeGI*; genome-wide decrease in methylation levels; and sexual distinct regulatory networks of smRNAs and their targets. However, the first mechanism was inconsistent in the monoecious system; *MeGI* expression and *smMeGI* accumulation were not significantly different between M_F and M_M (Fig. 2). This is inconsistent with the pattern in monoecious hexaploid *D. kaki*, which has lower *smMeGI* levels and higher *MeGI* levels in the female flower buds of genetically male plants [10]. Considering the similar *MeGI* expression in monoecious (both male and female floral buds) and androecious (male floral buds) plants (Fig. 2F), monoecious *D. oleifera* may develop gynoecia independently of *MeGI* regulation.

The mechanism that underlies gynoecia development in monoecious plants may be global regulation of DNA methylation (Fig. 3). Treatment of male flower buds with the DNA methylation inhibitor zebularine and 5-azacytidine induces pistil development and reduces pollen fertility in some *D. kaki* cultivars [10, 28]. The DNA demethylase gene *ROS1* [18] was upregulated in hermaphroditic floral buds compared with male floral buds in andromonoecious *D. oleifera* (Table S20), which may explain the genome-wide decrease in methylation level in hermaphroditic floral buds compared with male floral buds*.*

We also identified several putative key relationships between miRNA expression and target expression. Decreased expression of *GAMYBs* (evm.model.Chr13.1162 and evm.model.Chr11.1032) and increased expression of putative miRNA regulators were identified in female (or hermaphroditic) floral buds compared with male floral buds. *GAMYB* is a *trans*-activator of GA signalling [29, 30] and functions in flower development [31, 32]. The upregulation of *GAMYB* in male floral buds implies that GA signalling promotes the development of androecious tissues, consistent with our previous findings that GA promotes the male function in monoecious [33, 34] and andromonoecious [12] *D. kaki*. Furthermore, *SPL* family genes (evm.model.Chr14.920, evm.model.Chr7.129, evm.model.Chr7.171, and evm.model.Chr12.765), *JMJ25*, and *GRFs* were commonly activated in female tissues, presumably through miRNA regulation. *SPL* family genes have diverse functions in plant development [35]; one of these genes acts a direct upstream activator of *LEAFY*, *FRUITFULL*, and *APETALA1* to control the timing of flower formation [36]. *JMJ25* is a histone H3K9 demethylase gene that reportedly affects DNA methylation [37]. *GRFs* are plant-specific transcription factors, and the *miR396/GRF* regulatory network is required for the proper development of the pistil in *Arabidopsis* [38, 39]. The functions of these genes in model plants and their expression patterns in *D. oleifera* were consistent with the working hypothesis regarding sexual expression in *D. oleifera*.

Expression of heat stress TF B-3 (*HSFB3*) was reported to be firmly correlated with abiotic and biotic stress [40]. *HSFB3* was sharply higher expressed in male floral buds than that in female, suggesting that when plants were suffering from abiotic or biotic stress, they tended to bear male flowers. Thus, protection against abiotic or biotic stress contributes to the feminising scenario in *D. oleifera*.

It is worth noting that samples used for methylome and transcriptome analyses were obtained in mid-Apil, when the pistil or stamen primordia inside persimmon floral buds were alternatively arrested, leading to the final sex expression [9]. Thus, the feminising scenario uncovered at this developmental stage should be a part of sex determination system regulated by the upstream genetic factors.

### Genetic factors linked to the dissolution of dioecy

The monoecious phenotype in *D. oleifera* was unique and could not be well explained by known mechanisms. Therefore, we evaluated the genetic mechanisms that underlie the sexuality of monoecious and andromonoecious types. The candidate region for the monoecious trait on chromosome 7 included a cluster of seven *DUF247* genes (Fig. 6). The Y-specific dominant female suppression gene, *SOFF*, in dioecious *Asparagus officinalis* is a member of this gene family [41]. In *A. officinalis*, knockout of the *SOFF* gene converts males to hermaphrodites, knockout of the Y-specific male-promoting *aspTDF1* converts males to neuters, and knockout of both *TDF1* and *SOFF* converts males to females [42]. A *DUF247* family gene was identified as a male component of the self-incompatibility *S*-locus in perennial ryegrass [43, 44]. We detected clusters of duplicated *DUF* family genes in at least five loci in the *D. oleifera* genome (data not shown), implying functional divergence. The monoecious determinant, as well as its molecular genetic control, must be identified in subsequent studies. Although a very strong signal was obtained for chromosome 7, it could not explain all monoecious phenotypes (Fig. 6B), implying that other loci and environmental factors affect female development.

The development of hermaphroditic flowers in dioecious systems because of mutations at the sex-determining locus has been observed in grape and papaya [26, 27, 45]. In contrast, our GWAS approach for the differentiation of hermaphroditic floral buds yielded candidate regions on chromosomes 2, 11, and 14, but not on chromosome 4 (which has the sex-linked region). We also observed decreased expression of the feminising gene *MeGI* in hermaphroditic flower buds (Fig. 2F). Therefore, the establishment of hermaphroditic flower development in *D. oleifera* is independent of direct activation/inactivation of the genetic regulation of sex dimorphism through the existing *OGI*/*MeGI* system, as implied in work regarding *D. kaki* [7, 12].

Masuda et al. (2022b) reported that *DkRAD* regulates gynoecia formation in hermaphroditic flowers of hexaploid *D. kaki* [7]. This may also function in *D. oleifera* because the expression of the *DkRAD* homologue was higher in female and hermaphroditic tissues than in male tissues (Fig. S44), as in *D. kaki*. One discrepancy compared with the work of Masuda et al. (2022b) [7] is that our study revealed many diploid *D. oleifera* plants bearing hermaphroditic flowers, whereas Masuda et al. regarded the hermaphrodite mechanism in *Diospyros* as polyploid species-specific. Our results indicate that the evolution of hermaphroditic flower development in *Diospyros* is not ploidy-dependent; however, considering the similarity in sexual expression between *D. kaki* and *D. oleifera*, as well as their close phylogenetic relationship [46], a common evolutionary event and mechanism presumably led to sex expression diversity in both species. Further genetic analyses of sex expression in *D. kaki* and *D. oleifera* are needed.

### Summary and future perspectives

**Fig. 10:**
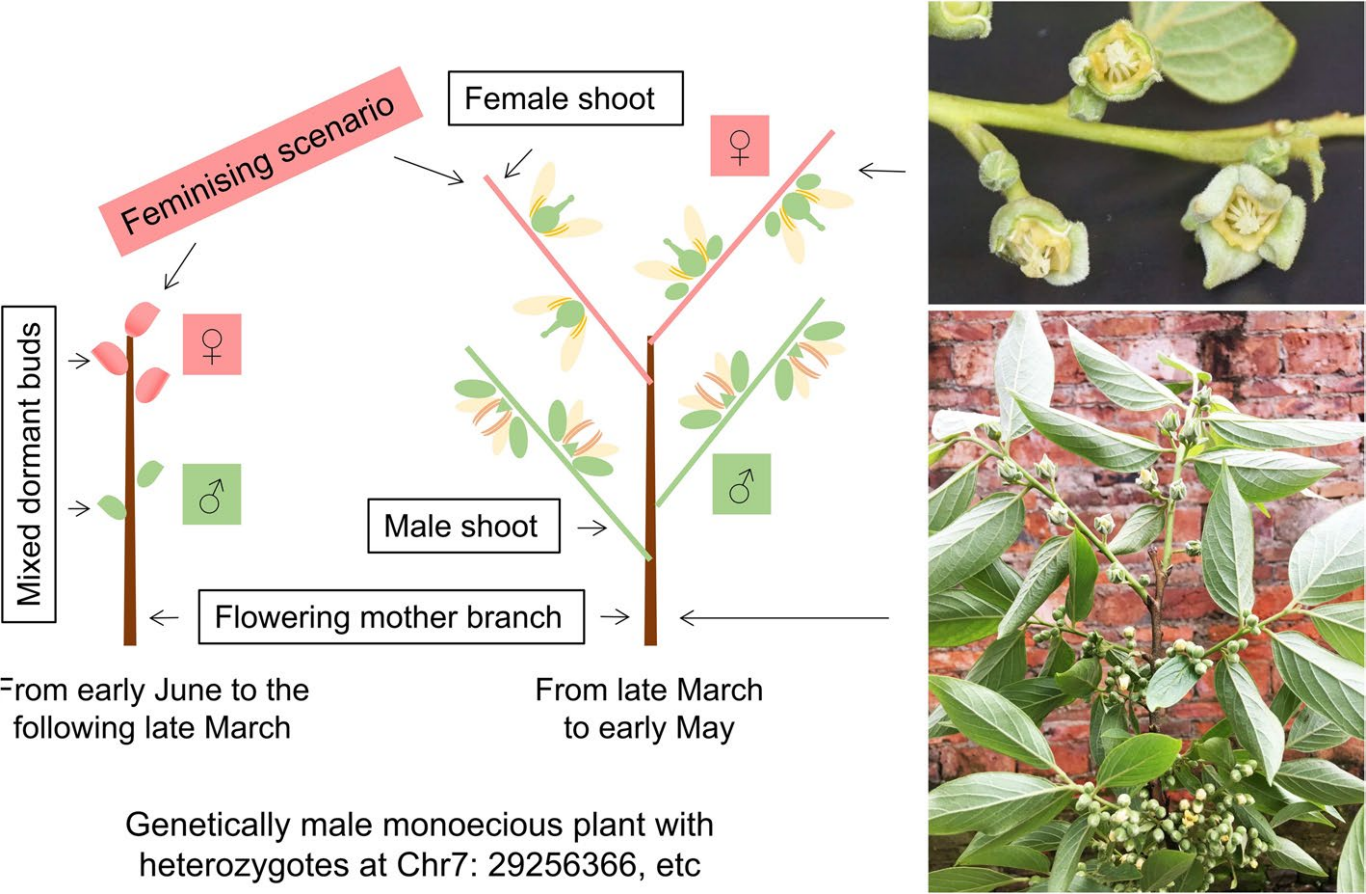
Schematic of the feminising scenario in monoecious *D. oleifera*

Based on our findings for *D. oleifera*, female shoots mainly develop from mixed dormant buds that developed on the tips of the flowering mother branches in monoecious plants which are genetically male with *OGI*. In contrast, male shoots mainly develop on the basal parts of the mother branches (Fig. 10). Floral primordia in *D. oleifera* are initiated in early summer, then experience a long dormant period until the following spring to break the buds [9]. Therefore, dormant floral buds on the tips of the flowering mother branches are more likely to activate the feminising scenario under natural conditions (Fig. 10). The same pattern was observed in cultivated hexaploid *D. kaki* (Fig. S45). An investigation considering arrangement of sex within flowering mother branches will further expand the understanding of physiological and molecular basis of sex expression in *Diospyros*.

## Conclusions

Co-sexual expression (monoecy and hermaphroditic development), previously thought to be polyploid-specific in *Diospyros* species, was identified in the diploid *D. oleifera* historically. We characterized potential genetic mechanisms that underlie the dissolution of dioecy to monoecy and andro(gyno)monoecy, based on multiscale genome-wide investigations of 150 accessions of *Diospyros oleifera*. We found all co-sexual plants, including monoecious and andro(gyno)monoecious individuals, possessed the male determinant gene *OGI*, implying the presence of genetic factors controlling gynoecia development in genetically male *D. oleifera*. In both single- and co-sexual plants, female function was expressed in the presence of a genome-wide decrease in methylation levels, along with sexually distinct regulatory networks of smRNAs and their targets. Furthermore, a genomic region and a *DUF247* gene cluster strongly associated with the monoecious phenotype and several regions that may contribute to andromonoecy were identified. Collectively, our findings demonstrate stable breakdown of the dioecious system in *D. oleifera*, presumably also a result of genomic features of the Y-linked region.

## Methods

### Plant material and phenotyping

A *D. oleifera* collection from Guilin, Guangxi Zhuang Autonomous Region, China, was evaluated for sexual expression in the 2019 and 2021 seasons. Two hundred eight *D. oleifera* trees were found in the natural population. In *D. oleifera*, the flower sex on a single shoot is uniform. Thus, for simplicity and accuracy, large flowering mother branches (approximately 1.5 m in height × 1.5 m in width) containing ≥ 20 flowering shoots (each uniformly bearing female, male, or hermaphroditic floral buds) were used to calculate the proportions of female, male, and hermaphroditic shoots in monoecious, androgynomonoecious, and andromonoecious trees. At least five large flowering mother branches of each tree were selected; the highest female and hermaphroditic shoot proportions of the large flowering mother branches were used for GWAS as an indication of feminising ability on the co-sexual tree.

### Reference sequence construction

The female plant ‘*D. oleifera* 1’ was used to optimise the published version of the *D. oleifera* genome [47] using a BioNano optical mapping-assisted assembly (Method S2), resulting in a new *D. oleifera* main genome. This genome was deposited in figshare (https://doi.org/10.6084/m9.figshare.20101664.v3) [15]. The male-specific region (the MSR) was absent in this genome. Thus, the resequencing reads of 14 androecious, 4 andromonoecious, 15 monoecious, 7 androgynomonoecious, and 2 pseudo-monoecious *D. oleifera* individuals (detail information of resequencing reads was introduced in the following text) were mapped to the *D. oleifera* main genome with the Burrows-Wheeler Aligner (BWA) mem option and the paired-end model [48]. The unmapped reads were extracted using SAMtools [49], and they were assembled using SoapDenovo [50] to construct the male-unmapped sequences (Methods S3-S4), which was deposited in figshare (https://doi.org/10.6084/m9.figshare.20407386.v1) [51]. The *D. oleifera* main genome [15], male-unmapped sequences [51], and the *D. oleifera* chloroplast genome [46] were combined as a reference genome for methylome, whole-transcriptome, and resequencing analyses.

### Methylome and transcriptome analyses

Floral bud and immature stem tissues of flowering shoots were sampled in mid-April (April 15–17th), 2019, which is a key period for the differentiation of flower sex types (Method S6). Those samples (Table 2) were used for the whole-genome bisulphite sequencing, transcriptome sequencing, and small RNA (smRNA) sequencing. All reads obtained were mapped to the combined reference *D. oleifera* genome. Details of library construction, sequencing, and analysis are provided in Methods S7-S9.

**Table 2:** Classification of samples used for methylome detection and transcriptome analysis

Sex phenotype of a tree	Groups	Samples within each group
**Classification of samples used for methylome detection**		
Gynoecious (*OGI*^−^)	G_F (gynoecious_female floral buds)	11F; 21F; 186F
	G_S (gynoecious_stems of immature flowering shoots)	11S; 21S; 186S
Androecious (*OGI*^+^)	A_M (androecious_male floral buds)	13 M; 65 M; 188 M
	A_S (androecious_stems of immature flowering shoots)	13S; 65S; 188S
Monoecious (*OGI*^+^)	M_F (monoecious_female floral buds)	108F; 168F
	M_M (monoecious_male floral buds)	108 M; 168 M
	M_S (monoecious_stems of immature flowering shoots)	108S; 168S
Andromonoecious (*OGI*^+^)	AM_M (andromonoecious_male floral buds)	206 M with three biological replicates
	AM_H (andromonoecious_hermaphroditic floral buds)	206H with two biological replicates
	AM_L (andromonoecy_leaf)	206L with three biological replicates
Pseudo-monoecious (*OGI*^−^)	PM_SF (pseudo-monoecy_solitary female floral buds)	PM_SF
	PM_MF (pseudo-monoecy_middle floral buds obtained from the three flower cymes)	PM_MF
	PM_LF (pseudo-monoecy_lateral floral buds obtained from the three flower cymes)	PM_LF
	PM_S (pseudo-monoecy_stems of immature flowering shoots)	PM_S
**Classification of samples used for transcriptome analysis**		
Gynoecious (*OGI*^−^)	G_F (gynoecy_female floral buds)	11F; 21F; 186F
	G_S (gynoecy_stems of immature flowering shoots)	11S; 21S; 186S
Androecious (*OGI*^+^)	A_M (androecy_male floral buds)	13 M; 65 M; 188 M
	A_S (androecy_stems of immature flowering shoots)	13S; 65S; 188S
Monoecious (*OGI*^+^)	M_F (monoecy_female floral buds)	108F; 136F; 168F
	M_M (monoecy_male floral buds)	108 M; 136 M; 168 M
Andromonoecious (*OGI*^+^)	AM_M (andromonoecy_male floral buds)	206 M with three biological replicates
	AM_H (andromonoecy_hermaphroditic floral buds)	206H with three biological replicates
Pseudo-monoecious (*OGI*^−^)	PM_SF (pseudo-monoecy_solitary female floral buds)	PM_SF
	PM_LF (pseudo monoecy_lateral floral buds obtained from the three flower cymes)	PM_LF

The smRNAs-Seq reads were also mapped to the *OGI* sequence from the *D. lotus* genome [16], *MeGI* sequence from the *D. oleifera* reference genome, and the ‘*Kali*’ sequence reported by [10], using the method established by Akagi et al. (2016a) [10]*.* Here, the smRNAs-Seq reads mapped onto the *MeGI* gene body were referred to as *smMeGI*. The accumulation levels of *smMeGI* and each fragment were recorded as reads per million reads. Two independent-samples T test (SPSS, Inc, Chicago, IL, USA) was used to determine the significant difference between female (or hermaphroditic) and male floral buds (or stems) in each sexual systems.

### Resequencing analysis

A set of 150 *D. oleifera* (Table 1; Table S13) was selected from the collection and used for resequencing analysis. Short read resequencing (PE150) by the Illumina NovaSeq 6000 platform yielded 3.03 Tb of raw data with ~ 20-fold genomic coverage for each sample. All reads were firstly mapped to the *D. lotus OGI* genomic sequence and the *D. lotus* the MSR sequence [16] using the Bwa mem option and the paired-end model to check whether each *D. oleifera* individual was *OGI*-positive or not. Subsequently, all the reads were mapped the combined *D. oleifera* reference genome using the Bwa mem option and the paired-end model. SAMtools and the Genome Analysis Toolkit (version 2.4–7-g5e89f01) were used to label SNPs and insertion-deletions (indels). Polymorphisms that matched the following four criteria were filtered out: > 2 alleles, variants beyond the read depth between half and twice the genome-wide average, missing rates ≥ 0.25, and minor allele frequency < 0.05. The linkage disequilibrium (LD) was evaluated using the pairwise squared Pearson’s correlation coefficient (r^2^) calculated by PLINK version 1.9 [52]. LD pruning was conducted by a standard method (PLINK –indep 50 5 2).

Using the filtered variant sets, the following analyses were conducted. First, the familial relationships and sample uniqueness were evaluated based on the PI_HAT value computed by PLINK. Then the population structure of the 150 trees was estimated using principal component analysis (PCA) in EIGENSTRAT software [53]. The maximum-likelihood phylogenetic tree was constructed using MEGA-X [54] with 1000 replicates using the following parameters: gaps/missing data, partial deletion; site coverage cut-off, 90%; general time reversible model; and rates among sites, uniform.

### GWAS

GWAS was performed using the linear mixed model in the R package rrBLUP [55]. A kinship (K) matrix (generated with the A.mat function of rrBLUP) was included in the linear mixed model, along with 6 principal components (PCs) for 90 individuals with male production (Fig. S40B) and 4 PCs for all 150 individuals (Fig. S42). Bonferroni correction (corrected *P* < 0.05) was used to determine the genome-wide significance thresholds. The LD patterns surrounding GWAS peaks were visualised using the R package LDheatmap [56] for chromosome 7, and using Haploview (http://www.broadinstitute.org/haploview) for chromosomes 4, 2, 11, and 14. The regions with pairwise r^2^ > 0.5 were regarded as candidate LD blocks.

### Supplementary Information

**Additional file 1.**

**Additional file 2.**

**Additional file 3.**

**Additional file 4.**

## Data Availability

The raw sequence data of DNA resequencing, BS-seq, smRNA and BioNano molecules that support the findings of this study are openly available in the Genome Sequence Archive (GSA, https://ngdc.cncb.ac.cn/gsa/) with accession numbers of CRA007608, CRA007609, CRA007611, CRA007613, respectively. The raw sequence data of lncRNA that support the findings of this study are openly available in the Genome Sequence Archive (GSA, https://ngdc.cncb.ac.cn/gsa/) with an accession number of CRA007610 as reported by Mai et al. (2022) [57].

## Abbreviations

A_M: Androecious male floral buds
A_S: Andromonoecious stems of immature flowering shoots
AM_H: Andromonoecious hermaphroditic floral buds
AM_M: Andromonoecious male floral buds
BWA: Burrows-Wheeler Aligner
FPKM: Fragments per kilobase of transcript per million mapped reads
*GAM1*: *GAMYB*
*GRF6*: Growth-regulating factor 6
GWAS: Genome-wide association study
G_F: Gynoecious female floral buds
G_S: Gynoecious stems of immature flowering shoots
*HSFB3*: Heat stress TF B-3
indels: Insertion-deletions
*J*: MADS-box protein JOINTLESS
*JMJ25*: Lysine-specific demethylase
KEGG: Kyoto Encyclopedia of Genes and Genomes
LD: Linkage disequilibrium
*MFP1-1*: MAR-binding filament-like protein 1–1
miRNAs: MicroRNAs
the MSR: Male-specific region
*myb12*: Myb-related protein B
M_F: Monoecious female floral buds
M_M: Monoecious male floral buds
*OGI*^−^: *OGI*-Negative
*OGI*^+^: *OGI*-Positive
*ORCS-1A*: Origin of replication complex subunit 1A
PCA: Principal component analysis
PM_LF: Pseudo-monoecious lateral female floral buds
PM_SF: Pseudo-monoecious solitary female floral buds
*ROS1*: REPRESSOR OF SILENCING1
*smMeGI*: Accumulation patterns of smRNAs on *OGI* and *MeGI* regions
smRNA: Small RNA
SNPs: Single nucleotide polymorphisms
*SPL7*: Squamosa promoter-binding-like protein gene* 7*

## References

[1] Renner SS, Ricklefs RE (1995). Dioecy and its correlates in the flowering plants. Am J Bot.

[2] Weiblen GD, Oyama RK, Donoghue MJ (2000). Phylogenetic analysis of dioecy in monocotyledons. Am Nat.

[3] Renner SS (2014). The relative and absolute frequencies of angiosperm sexual systems: dioecy, monoecy, gynodioecy, and an updated online database. Am J Bot.

[4] Heilbuth JC (2000). Lower species richness in dioecious Clades. Am Nat.

[5] Kafer J, Marais GAB, Pannell JR (2017). On the rarity of dioecy in flowering plants. Mol Ecol.

[6] Badouin H, Velt A, Gindraud F, Flutre T, Marais GA (2020). The wild grape genome sequence provides insights into the transition from dioecy to hermaphroditism during grape domestication. Genome Biol.

[7] Masuda K, Ikeda Y, Matsuura T, Kawakatsu T, Tao R, Kubo Y, Ushijima K, Henry IM, Akagi T (2022). Reinvention of hermaphroditism via activation of a RADIALIS-like gene in hexaploid persimmon. Nat plants.

[8] Akagi T, Henry IM, Tao R, Comai L (2014). A Y-chromosome-encoded small RNA acts as a sex determinant in persimmons. Science.

[9] Li JR, Sun P, Han WJ, Li FD, Fu JM, Diao SF (2016). Morphological key period study on floral sex differentiation in pollination-constant and non-astringent persimmon ‘Zenjimaru’. Acta Horticulturae Sinica.

[10] Akagi T, Henry IM, Kawai T, Comai L, Tao R (2016). Epigenetic regulation of the sex determination gene *MeGI* in polyploid persimmon. Plant Cell.

[11] Wang L, Li H, Sun P, Suo Y, Han W, Diao S, Mai Y, Li F, Fu J (2021). Efects of plant growth regulators, soil moisture contents, and carbon/nitrogen ratios on sex diferentiation in Persimmon (*Diospyros kaki* Thunb.) Flowers. J Plant Growth Regul.

[12] Li H, Wang L, Mai Y, Han W, Suo Y, Diao S, Sun P, Fu J (2021). Phytohormone and integrated mRNA and miRNA transcriptome analyses and differentiation of male between hermaphroditic floral buds of andromonoecious *Diospyros kaki* Thunb. BMC Genomics.

[13] Henry IM, Akagi T, Tao R, Comai L (2018). One hundred ways to invent the sexes: theoretical and observed paths to dioecy in plants. Annu Rev Plant Biol.

[14] Masuda K, Akagi T, Tao R, Luo Z (2022). Sexual system and its evolution. The Persimmon Genome.

[15] Sun P, Fu JM. A BioNano optical mapping-assisted chromosomal genome assembly of *Diospyros oleifera*. *figshare*. Dataset. 2022a. 10.6084/m9.figshare.20101664.v3

[16] Akagi T, Shirasawa K, Nagasaki H, Hirakawa H, Tao R, Comai L, Henry IM (2020). The persimmon genome reveals clues to the evolution of a lineage-specific sex determination system in plants. PLoS Genet.

[17] Akagi T, Kawai T, Tao R (2016). A male determinant gene in diploid dioecious Diospyros, OGI, is required for male flower production in monoecious individuals of Oriental persimmon (D. kaki). Sci Hortic-Amsterdam.

[18] Gong Z, Morales-Ruiz T, Ariza RR, Roldan-Arjona T, David L, Zhu JK (2002). *ROS1*, a repressor of transcriptional gene silencing in *Arabidopsis*, encodes a DNA glycosylase/lyase. Cell.

[19] Kanehisa M, Goto S (2000). KEGG: Kyoto Encyclopedia of Genes and Genomes. Nucleic Acids Res.

[20] Kanehisa M (2019). Toward understanding the origin and evolution of cellular organisms. Protein Sci.

[21] Kanehisa M, Furumichi M, Sato Y, Kawashima M, Ishiguro-Watanabe M (2023). KEGG for taxonomy-based analysis of pathways and genomes. Nucleic Acids Res.

[22] Crossman A, Charlesworth D (2014). Breakdown of dioecy: models where males acquire cosexual functions. Evolution.

[23] Perbal MC, Haughn G, Saedler H, Schwarz-Sommer Z (1996). Non-cell-autonomous function of the *Antirrhinum* floral homeotic proteins *DEFICIENS* and *GLOBOSA* is exerted by their polar cell-to-cell trafficking. Development.

[24] Müller NA, Kersten B, Montalvão APL, Mähler N, Bernhardsson C, Bräutigam K, Lorenzo ZC, Hoenicka H, Kumar V, Mader M, Pakull B, Robinson KM, Sabatti M, Vettori C, Ingvarsson PK, Cronk Q, Street NR, Fladung M (2020). A single gene underlies the dynamic evolution of poplar sex determination. Nat plants.

[25] Cossard GG, Gerchen JF, Li X, Cuenot Y, Pannell JR (2021). The rapid dissolution of dioecy by experimental evolution. Current Biol.

[26] Massonnet M, Cochetel N, Minio A (2020). The genetic basis of sex determination in grapes. Nat Commun.

[27] VanBuren R, Zeng F, Chen C, Zhang J, Wai MC, Han J, Aryal R, Gschwend AR, Wang J, Na JK, Huang L, Zhang L, Miao W, Gou J, Arro J, Guyot R, Moore RC, Wang ML, Zee F, Charlesworth D, Moore PH, Yu Q, Ming R (2015). Origin and domestication of papaya Y^h^ chromosome. Genome Res.

[28] Wang L, Li H, Suo Y, Han W, Diao S, Mai Y, Wang Y, Yuan J, Ye L, Pu T, Zhang Q, Sun P, Li F, Fu J (2022). Effects of different chemicals on sexual regulation in persimmon (Diospyros kaki Thunb.) Flowers. Front Plant Sci.

[29] Gubler F, Kalla R, Roberts JK, Jacobsen JV (1995). Gibberellin-regulated expression of a myb gene in barley aleurone cells: evidence for Myb transactivation of a high-pI alpha-amylase gene promoter. Plant Cell.

[30] Haseneyer G, Ravel C, Dardevet M, Balfourier F, Sourdille P, Charmet G, Brunel D, Sauer S, Geiger HH, Graner A, Stracke S (2008). High level of conservation between genes coding for the GAMYB transcription factor in barley (Hordeum vulgare L.) and bread wheat (Triticum aestivum L.) collections. Theor Appl Genet.

[31] Gocal GFW, Poole AT, Gubler F, Watts RJ, Blundell C, King RW (1999). Long-day up-regulation of a *GAMYB* gene during *Lolium temulentum* inflorescence formation. Plant Physiol.

[32] Murray F, Kalla R, Jacobsen J, Gubler F (2003). A role for HvGAMYB in anther development. Plant J.

[33] Sun P, Li JR, Du GG, Han WJ, Fu JM, Diao SF, Suo YJ, Zhang Y, Li FD (2017). Endogenous phytohormone profiles in male and female floral buds of the persimmons (*Diospyros kaki* Thunb.) during development. Sci Hortic-Amsterdam..

[34] Li SZ, Sun P, Du GG, Wang LY, Li HW, Fu JM, Suo YJ, Han WJ, Diao SF, Mai YN, Li FD (2019). Transcriptome sequencing and comparative analysis between male and female floral buds of the persimmon (Diospyros kaki Thunb.). Sci Hortic-Amsterdam..

[35] Preston JC, Hileman LC (2013). Functional evolution in the plant *SQUAMOSA-PROMOTER BINDING PROTEIN-LIKE* (*SPL*) gene family. Front Plant Sci.

[36] Yamaguchi A, Wu MF, Yang L, Wu G, Poethig RS, Wagner D (2009). The microRNA-regulated SBP-Box transcription factor *SPL3* is a direct upstream activator of *LEAFY*, *FRUITFULL*, and *APETALA1*. Dev Cell.

[37] Fan D, Wang X, Tang X, Ye X, Ren S, Wang D, Luo K (2018). Histone H3K9 demethylase JMJ25 epigenetically modulates anthocyanin biosynthesis in poplar. Plant J.

[38] Omidbakhshfard MA, Proost S, Fujikura U, Mueller-Roeber B (2015). Growth-regulating factors (GRFs): a small transcription factor family with important functions in plant biology. Mol Plant.

[39] Liang G, He H, Li Y, Wang F, Yu D (2014). Molecular mechanism of microRNA396 mediating pistil development in *Arabidopsis*. Plant Physiol.

[40] Feder ME, Hofmann GE (1999). Heat-shock proteins, molecular chaperones, and the stress response: evolutionary and ecological physiology. Annu Rev Physiol.

[41] Harkess A, Zhou J, Xu C, Bowers JE, Van der Hulst R, Ayyampalayam S (2017). The asparagus genome sheds light on the origin and evolution of a young Y chromosome. Nat Commun.

[42] Harkess A, Huang K, Van der Hulst R, Tissen B, Caplan JL, Koppula A, Batish M, Meyers BC, Leebens-Mackb J (2020). Sex determination by two Y-linked genes in garden asparagus. Plant Cell.

[43] Manzanares C, Barth S, Thorogood D, Thorogood D, Byrne SL, Yates S, Czaban A, Asp T, Yang B, Studer B (2016). A gene encoding a DUF247 domain protein cosegregates with the S self-Incompatibility locus in perennial ryegrass. Molr Biol Evol.

[44] Thorogood D, Yates S, Manzanares C, Skot L, Hegarty M, Blackmore T, Barth S, Studer B (2017). A novel multivariate approach to phenotyping and association mapping of multi-locus gametophytic self-incompatibility reveals S, Z, and other loci in a perennial ryegrass (Poaceae) population. Front Plant Sci.

[45] Valleau WD (1915). Inheritance of sex in the grape. Am Nat.

[46] Fu JM, Liu HM, Hu JJ, Liang YQ, Liang JJ, Wuyun TN, Tan XF (2017). Five complete chloroplast genome sequences from *Diospyros*: genome organization and comparative analysis. PLoS ONE.

[47] Suo YJ, Sun P, Cheng HH, Han WJ, Diao SF, Li HW, Mai YN, Zhao X, Li FD, Fu JM (2020). A high-quality chromosomal genome assembly of *Diospyros oleifera* Cheng. GigaScience.

[48] Li H, Durbin R (2010). Fast and accurate long-read alignment with Burrows-Wheeler transform. Bioinformatics.

[49] Li H, Handsaker B, Wysoker A, Fennell T, Ruan J, Homer N, Marth G, Abecasis G, Durbin R (2009). 1000 genome project data processing subgroup. The sequence alignment/map (SAM) format and SAMtools. Bioinformatics.

[50] Li R, Zhu H, Ruan J, Qian W, Fang X, Shi Z, Li Y, Li S, Shan G, Kristiansen K, Li S, Yang H, Wang J, Wang J (2010). De novo assembly of human genomes with massively parallel short read sequencing. Genome Res.

[51] Sun P, Fu JM. The *Diospyros oleifera* heterozygous and male unmapped sequences. *figshare*. Dataset. 2022b. 10.6084/m9.figshare.20407386.v1.

[52] Slifer SH (2018). PLINK: Key Functions for data analysis. Curr Protoc Hum Genet.

[53] Price AL, Patterson NJ, Plenge RM, Weinblatt ME, Shadick NA, Reich D (2006). Principal components analysis corrects for stratification in genome-wide association studies. Nat Genet.

[54] Kumar S, Stecher G, Tamura K (2016). MEGA7: Molecular evolutionary genetics analysis version 7.0 for bigger datasets. Mol Biol Evol.

[55] Endelman JB (2011). Ridge regression and other kernels for genomic selection with R package rrBLUP. Plant Genome-US.

[56] Shin J, Blay S, McNeney B, Graham J (2006). LDheatmap: An R function for graphical display of pairwise linkage disequilibria between single nucleotide polymorphisms. J Stat Softw.

[57] Mai Y, Diao S, Yuan J, Wang L, Suo Y, Li H, Han W, Wang Y, Ye L, Liu Y (2022). Identification and analysis of MADS-box, WRKY, NAC, and SBP-box transcription factor families in Diospyros oleifera Cheng and their associations with sex differentiation. Agronomy-basel.

